# Femoral Fracture in Pregnancy: A Case Report and Review of Data from the Literature

**DOI:** 10.3390/life15040601

**Published:** 2025-04-04

**Authors:** Ștefan-Dragoș Tîrnovanu, Elena Cojocaru, Bogdan Veliceasa, Norin Forna, Adrian-Claudiu Carp, Bogdan Puha, Alexandru Filip, Awad Dmour, Dragoș-Cristian Popescu, Ovidiu Alexa, Sorana-Caterina Anton, Mihaela-Camelia Tîrnovanu

**Affiliations:** 1 Department of Orthopedics and Traumatology, “Grigore T. Popa” University of Medicine and Pharmacy, 700115 Iasi, Romania; stefan-dragos.tirnovanu@d.umfiasi.ro (Ș.-D.T.); bogdan.veliceasa@umfiasi.ro (B.V.); norin.forna@umfiasi.ro (N.F.); adrian-claudiu.carp@umfiasi.ro (A.-C.C.); bogdan.puha@umfiasi.ro (B.P.); alexandru-filip@umfiasi.ro (A.F.); awad.dmour@d.umfiasi.ro (A.D.); dragos.popescu@umfiasi.ro (D.-C.P.); ovidiu.alexa@umfiasi.ro (O.A.); 2 “Saint Spiridon” County Emergency Clinical Hospital, 700111 Iasi, Romania; 3 Department of Morphofunctional Sciences I, “Grigore T. Popa” University of Medicine and Pharmacy, 700115 Iasi, Romania; 4 Department Orthopedics and Traumatology, Clinical Rehabilitation Hospital, 700661 Iasi, Romania; 5 Department of Mother and Child Medicine, “Grigore T. Popa” University of Medicine and Pharmacy, 700115 Iasi, Romania; sorana.anton@umfiasi.ro (S.-C.A.); mihaela.tirnovanu@umfiasi.ro (M.-C.T.); 6 “Cuza Voda” Obstetrics-Gynecology Clinic Hospital, 700038 Iasi, Romania

**Keywords:** femoral shaft fracture in pregnancy, pregnancy-associated osteoporosis, multidisciplinary trauma management in pregnancy, intramedullary nailing during pregnancy, radiation safety in obstetric orthopedic surgery

## Abstract

Background: Orthopedic trauma during pregnancy is a rare yet complex medical challenge, impacting both maternal and fetal health. Among these, femoral fractures are particularly uncommon but require careful management to minimize maternal and fetal risks. Methods: We report the case of a 28-year-old woman, gravida 4, para 3, at 40 weeks of gestation, who sustained a left mid-femoral diaphyseal fracture following a low-energy fall. A multidisciplinary team approach, including obstetric, orthopedic, anesthetic, and neonatal specialists, was employed. Preoperative imaging by X-ray was performed under lead-apron protection. The patient underwent an emergency C-section, followed by closed reduction and internal fixation with an intramedullary nail. Results: The surgical intervention was successful, with minimal radiation exposure. Postoperative management included thromboprophylaxis, calcium, vitamin D supplementation, and physiotherapy. The patient recovered well, achieving fracture healing within three months. Postpartum bone density assessment was recommended, suspecting pregnancy- and lactation-associated osteoporosis. Conclusions: Managing femoral fractures during pregnancy necessitates a balance between maternal and fetal well-being. A collaborative, multidisciplinary approach ensures optimal outcomes. Early surgical intervention, proper radiation precautions, and postpartum bone health assessment are crucial in these cases. Further research is needed to understand risk factors and preventive strategies for pregnancy-associated osteoporosis.

## 1. Introduction

Trauma sustained during pregnancy can trigger uncertainty and anxiety for the patient and orthopedic surgeon alike. In addition, pregnancy and trauma are complex situations with important implications for the health of both the mother and fetus [1]. The management of a pregnant trauma patient requires a multidisciplinary approach, with the need to balance the well-being of both the mother and the fetus. Challenges include minimizing radiation exposure during diagnostic procedures, selecting appropriate surgical timing, and using medications that are safe for pregnant patients and fetuses [2].

Although the causes of orthopedic traumas vary, pregnant women and their unborn babies are most vulnerable to injuries in motor vehicle crashes [1]. Another cause is partner violence or falls from the same level.

Orthopedic trauma in pregnancy is a rare event, with an incidence of 1–6% [3,4]. It has been observed that the presence of ankle fractures tends to occur most frequently in the second and third trimesters of pregnancy [5]. Femoral fractures in pregnancy are a rare complication, with an incidence of approximately 1% of all pregnancies [6]. Hip disorders in pregnancy can encompass transient osteoporosis or osteonecrosis, with fragility fractures or significant femoral head collapse occurring in rare cases. Notably, research has documented a fracture incidence of 18.2% among individuals diagnosed with transient osteoporosis during pregnancy [7]. A 2012 study indicates that almost 70% of women developed pregnancy and lactation-associated osteoporosis (PLO) during their first pregnancy [8].

Statistical comparisons between pregnant patients with orthopedic trauma and those without trauma highlight significant differences in perinatal outcomes. Trauma-affected pregnancies show a higher incidence of preterm birth, with 17% delivering between 24 and 33 weeks of gestation compared to 3% in the non-trauma group, and 31% giving birth before 37 weeks, also markedly higher than the 3% observed in non-trauma cases. Additionally, the rate of cesarean delivery is elevated by 15% in this population, and hospital admission frequently results in immediate delivery in 34% of trauma cases, compared to 13% in non-trauma pregnancies [9].

The bone lesions in pregnancy need to be managed by a mother and fetus orthopedic trauma care center if possible. A multidisciplinary approach must be taken [10,11]. Knowledge of maternal physiology and anatomy, radiation procedures and risks, teratogenic agents (infections, chemicals, and drugs), and proper surgical techniques can significantly improve maternal outcomes without endangering fetal health.

Surgery should be delayed, if possible, until after delivery of the newborn to minimize any risk of harm. Many fractures during pregnancy can be managed conservatively based on the fracture pattern and displacement. Sometimes, the fetus may be delivered before fracture fixation if it is near term. Fractures should be treated in the same way as in a non-pregnant patient. The use of minimally invasive techniques of fracture reduction and fixation or other techniques dependent on intraoperative imaging is not recommended for pregnant patients [12].

When planning the treatment of a pregnant patient with orthopedic trauma, the surgeon must be aware of the risks of miscarriage, preterm labor, placental abruption, preterm rupture of membranes, and fetal demise. Some reports estimate that the risk of intrauterine fetal demise (IUFD) is as high as 40.1%, depending on the location of the fracture [13]. Pelvic and acetabular fractures convey the highest morbidity and mortality [14,15].

## 2. Case Report

We report the case of a 28-year-old woman, gravida 4, para 3, at 40 weeks of gestation, who was brought to the Emergency Department of ’Sf. Spiridon’ University Hospital Iasi after sustaining a left thigh injury due to a fall from ground level. In her obstetrical history, prior to the present trauma during her third pregnancy, the patient had two previous deliveries and one abortion. The patient reported no additional medical problems or previous surgical history.

She was evaluated by a trauma surgeon. The physical examination revealed local deformation, edema with local swelling, and pain in the middle third of the thigh upon mobilization. Palpation identified localized pain, bone crepitus, and complete functional incapacity. She was without any open wounds or other complaints or recent traumatic marks on the pelvis or lower limb. She was neurovascularly intact.

An obstetric consultation was requested to assess the status of pregnancy. This is a must for the initial assessment, stabilization, and subsequent management of a pregnant trauma patient. The patient did not have painful uterine contractions or hypogastric pain. Clinical examination revealed a long and closed cervix. The uterine tone was normal. The fetus was monitored with cardiotocography and high-resolution real-time ultrasonography to provide information on fetal motion, heart rate, and placental aspect. The assessment with sonography showed good fetal motion and fetal indices.

**Figure 1:**
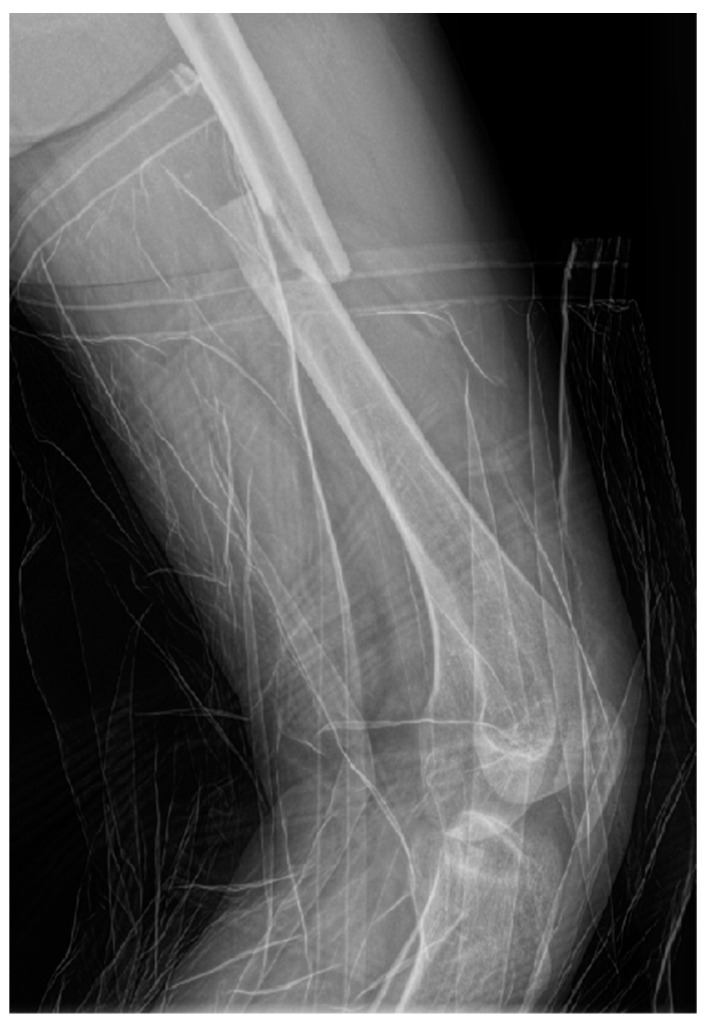
Profile X-ray of the left thigh revealing a fracture of the middle third of the femur with translation of the fragments.

Radiographs (X-ray) of the left thigh were performed, under lead-apron protection on the abdominopelvic region, with the consent of the obstetrician. The radiation dose used during the X-ray was minimal. The total preoperative and intraoperative radiation dosage was 135.2 mGy. The diagnosis was a left mid-femoral diaphysis fracture with displacement (Figure 1).

Blood tests were performed, revealing notable abnormalities in laboratory parameters. The patient exhibited hypocalcemia, with a total serum calcium level of 8.34 mg/dL, an elevated white blood cell count of 14.67 × 10^3^/μL, and mild anemia, indicated by a hemoglobin level of 9.9 g/dL.

**Figure 2:**
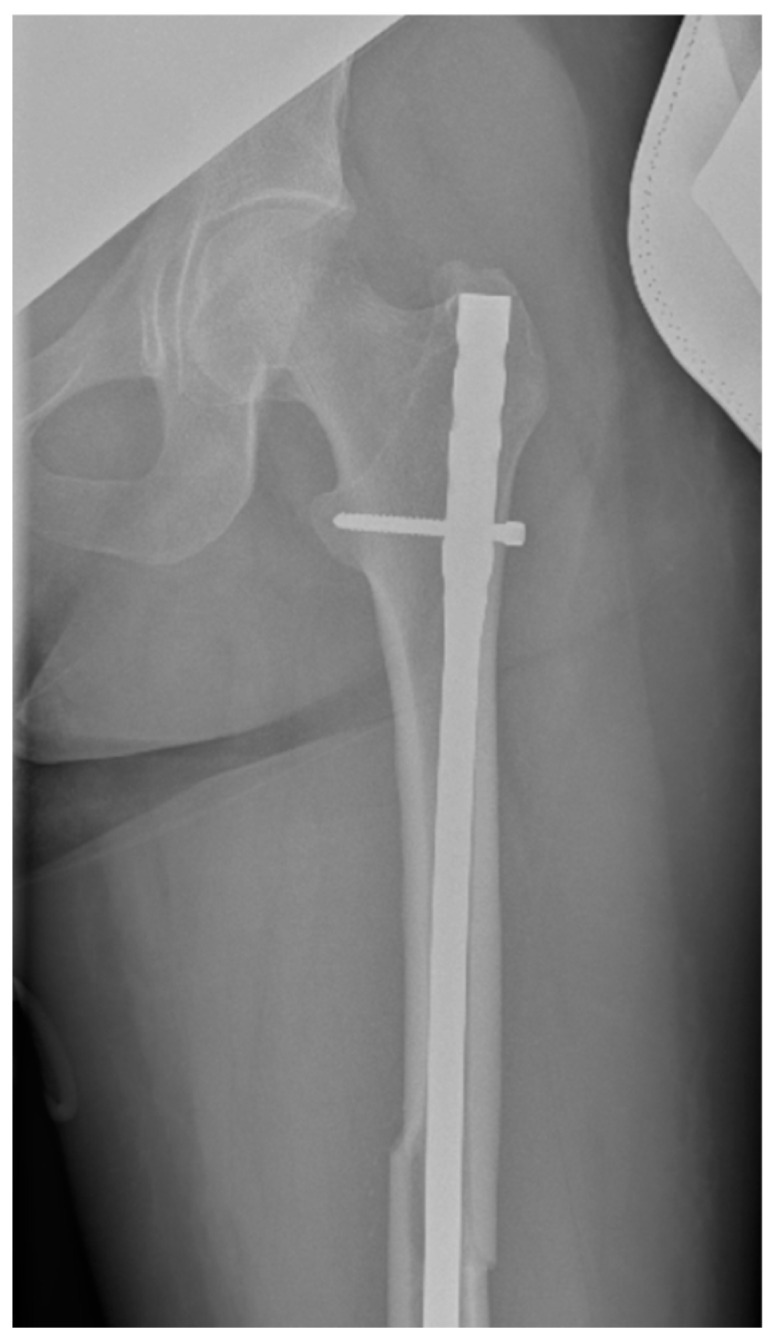
Postoperative anteroposterior view of the left thigh—proximal pole of the intramedullary anterograde nail fixed with one screw.

**Figure 3:**
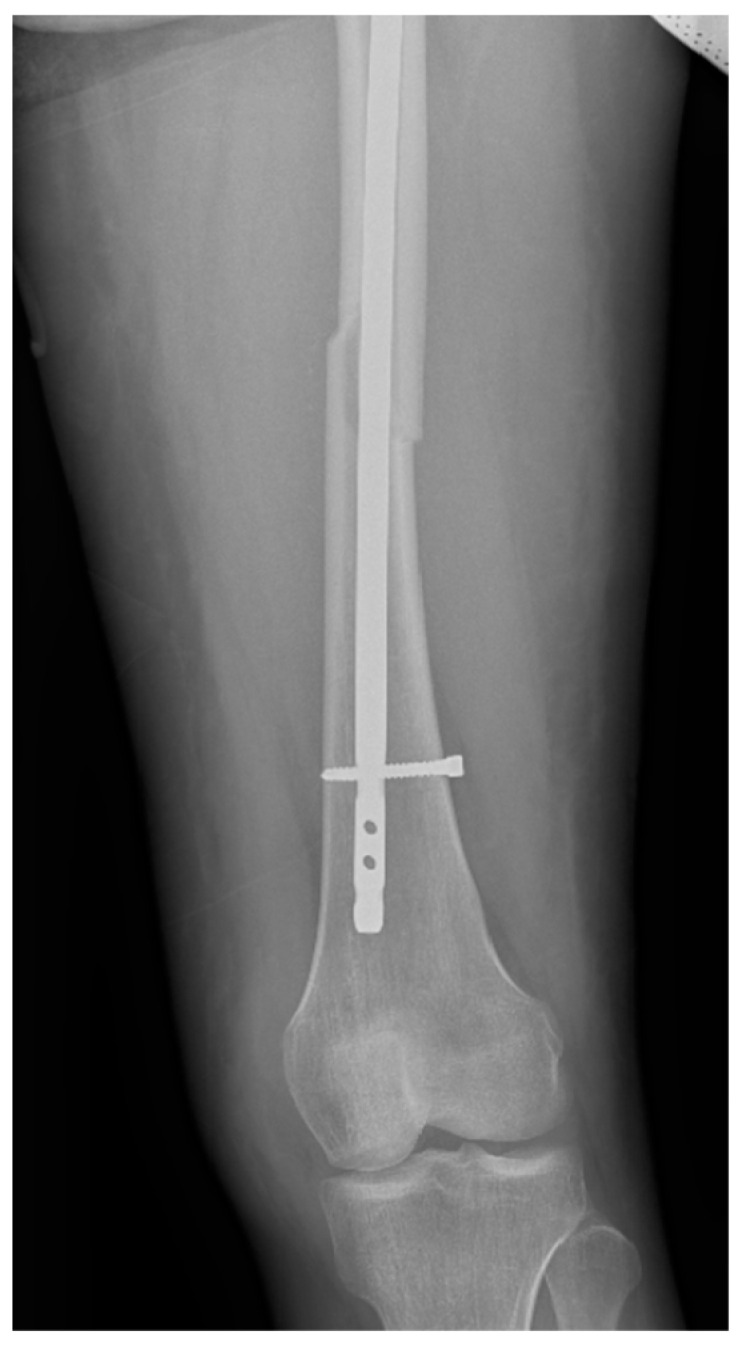
Postoperative anteroposterior view of the left thigh—distal pole of the intramedullary anterograde nail fixed statically with one screw.

Surgical intervention was performed by a multidisciplinary team: an anesthetist, an obstetrician, a neonatologist, and an orthopedist. She underwent spinal anesthesia utilizing 0.5% bupivacaine, with the extraction of a healthy female baby, weighing 3200 g, with an Apgar score of 9 by C-section and bilateral tubal ligation at the request of the patient, followed by a successful fracture repair under intraoperative C-arm fluoroscopy. The surgical intervention for the fracture of the femur consisted of closed reduction and osteosynthesis with an anterograde intramedullary nail locked proximally and distally with a screw. The postoperative X-ray control revealed good fracture reduction and correct placement of the osteosynthesis material (Figure 2 and Figure 3).

The postoperative progress of the patient was favorable under antibiotic protection and pain medication. Postoperative management included treatment with antithrombotic medication—low-molecular-weight heparin (LMWH) (Enoxaparin 40 mg × 2 daily)—and analgesics. Administration of enoxaparin started 48 h postoperatively. She was discharged 5 days after the surgery. The patient was cared for on an outpatient basis with the help of a walker. She continued the administration of low-molecular-weight heparin (LMWH) for 30 days, and supplements with calcium and vitamin D were recommended. Because PLO was suspected, we recommended not breastfeeding, but she was not compliant. Physiotherapy was needed. She was advised to use crutches for mobilization and avoid bearing weight on the left leg for 30 days. During this time, she was also recommended to start active-assisted and active range of motion exercises for the hip and knee. Her fracture healed satisfactorily in three months. Dual-energy X-ray absorptiometry (DXA) was recommended to assess bone density status after discharge from the hospital with revaluation after 1 year.

We consider that a collaborative team of well-trained specialists is essential for optimal patient and fetus outcomes.

## 3. Discussion

In general, pregnancy is a good state for the bones because a pregnant woman has very high levels of estrogen, which has an action for building bones. In the body, bones are constantly being built by osteoblasts and broken down by osteoclasts. Estrogen helps regulate this cycle [16]. During pregnancy, there is an increase in calcitriol production in the maternal kidneys, possibly in response to an increase in prolactin and placental lactogen. Increased calcitriol leads to enhanced maternal absorption of phosphorus and calcium by the intestines to match the increased fetal calcium demand [17]. In the third trimester of pregnancy, the fetal bone skeleton necessitates a total of 30 g of calcium (the equivalent of 250–300 mg daily calcium) provided by the mother [18].

During pregnancy, increased calcium intake is commonly recommended, either through dietary sources or supplementation, as advised by obstetricians. This is particularly beneficial given that peak bone mass is typically achieved by the late 20s to early 30s. While calcium supplements can help meet daily requirements, dietary sources such as dark leafy greens (e.g., spinach, kale, bok choy) and chia seeds provide a natural and bioavailable alternative. Since calcium absorption occurs in a limited segment of the small intestine, excessive intake at once—especially from supplements—may reduce overall absorption efficiency, limiting its benefits for bone health. Vitamin D is crucial for calcium absorption in the intestine, supporting bone development and skeletal integrity. Additionally, it plays a vital role in muscle function and regulates calcium homeostasis, which is essential for optimal neuromuscular and immune system function. The primary source of vitamin D for pregnant women is sunlight exposure; however, when endogenous production is insufficient, supplementation may be necessary to maintain adequate levels and support both maternal and fetal health. Vitamin D levels must be tested during pregnancy.

Increased bone turnover is quite obvious during pregnancy and lactation. In prospective longitudinal studies, the ranges of body mass density (BMD) decrease by skeletal location with 1–2% for the forearm, 1% for total hip, 4% for trochanter, 1–8% for femoral neck, and 1–9% for lumbar spine [19,20].

Research indicates that postpartum bone loss commonly occurs, regardless of whether a woman breastfeeds. This phenomenon is primarily attributed to a decline in estrogen levels combined with an increase in parathormone-related protein (PTHrP), which influences bone metabolism and resorption during this period. However, bone loss in this context is generally reversible after childbirth. The most significant loss of trabecular bone occurs in the early postpartum period, but bone mass is gradually regained after weaning. Engaging in physical activity post-weaning helps accelerate the recovery of bone mass [21,22].

On the other hand, during breastfeeding, women lose a usual daily amount of 210 mg of calcium. This means that after the first six months of breastfeeding, every woman loses a quantity of calcium four times more than that of the pregnancy period [23].

Bone mass generally replenishes following the resumption of the menstrual cycle. However, in rare instances, pregnancy- and lactation-associated osteoporosis (PLO) may significantly increase susceptibility to fractures, particularly in the hip or spine, even in the absence of significant trauma or impact. Nordin and Roper reported it for the first time in 1955 [24]. A common symptom of PLO is excruciating pain in the back or wherever the fracture has occurred [25].

Some women may begin their pregnancy with a lower bone density due to a pre-existing condition [26]. Risk factors for PLO include low body mass index, a family history of osteoporosis [27], a personal history of disordered eating or the female athlete triad; a digestive disease such as Crohn’s or inflammatory bowel disease, which can affect the absorption of calcium; or a bone disorder such as osteogenesis imperfecta. Women with these issues may benefit from talking to an endocrinologist and a nutritionist before becoming pregnant so they can plan to protect their bones as much as possible.

Other risk factors for PLO include maternal age above 30 years [28,29], smoking [30,31,32], low BMI, and vitamin D deficiency (level under 50 nmol/L).

The etiology of pregnancy- and lactation-associated osteoporosis (PLO), also referred to as transient osteoporosis of pregnancy, remains unclear. Affected individuals may experience increased bone fragility, leading to fractures—primarily in the spine or hip—either during pregnancy or postpartum, even in the absence of significant trauma. While these fractures can be painful and functionally debilitating, recovery generally occurs within a relatively short timeframe, and recurrence in subsequent pregnancies is uncommon. The underlying susceptibility to this rare condition remains poorly understood, warranting further investigation. Certain women may enter pregnancy with low bone density due to pre-existing conditions, medication use, or lifestyle factors, and the physiological increase in bone metabolism during gestation may exacerbate skeletal stress. Additionally, some pregnant individuals require heparin therapy for conditions such as antiphospholipid syndrome or thrombophilia, which has been linked to an increased risk of pregnancy-related osteoporosis. However, the benefits of anticoagulation for maternal and fetal survival outweigh the potential bone health risks. Other potential risk factors include a personal or family history of osteoporosis, previous fractures from minor trauma, loss of height (>3 cm), smoking, excessive alcohol consumption, sedentary lifestyle, and various medical conditions (e.g., diabetes, rheumatoid arthritis, systemic lupus erythematosus, chronic kidney or liver disease, endocrine disorders). Additionally, long-term use of certain medications, including glucocorticoids, antiepileptic drugs, some antipsychotics, and hormonal contraceptives, may contribute to bone loss. We consider that bariatric surgery must be added to this list because the number of cases that perform this type of intervention is increasing among young females of reproductive age and might impair their bone status. It increases the risk of fracture due to malabsorption, vitamin D deficiency, reduced bone formation caused by lower body weight, and gut-derived calciotropic hormone anomalies [33].

Some conditions that can favor PLO are strictly related to pregnancy, like glucocorticoid use in pregnancy, tocolysis with MgSO4 [34], gestational diabetes, anemia, in vitro fertilization, twin pregnancy, and postpartum thyroiditis [35,36].

The etiology of the femur fracture for our patient was presumed to be osteoporotic in origin, secondary to smoking, associated maybe with low levels of calcium and vitamin D. However, serum vitamin D levels were not assessed as part of the initial laboratory workup. Also, our patient was para 3 at 27 years, and some studies show that increased parity is correlated with the development of menopausal osteoporosis [37,38], indirect evidence of the loss of bone mass during pregnancy. The age of our case was 27 years old, younger than the risk age of 30 years reported in the literature. Low pre-pregnancy BMI might induce a lower bone mass density after birth [39]. Our patient had normal weight.

The available data on transient osteoporosis remains inconclusive, as it rarely presents with clinical symptoms during pregnancy or the postpartum period. However, when additional risk factors are present, osteoporosis may significantly complicate pregnancy, as observed in this case. This highlights the importance of recognizing and considering this diagnosis, particularly in younger patients who may not typically be perceived as at risk. For this reason, there are a few simple steps to help support bone health (Table 1).

Radiation exposure during fracture management in pregnant patients is a significant concern for both physicians and expectant mothers. Many orthopedic interventions necessitate imaging modalities, including plain radiographs, CT scans, and intraoperative C-arm fluoroscopy, each requiring specific safety measures to minimize fetal risk. The period of greatest fetal vulnerability to ionizing radiation, particularly in terms of central nervous system development, occurs between 8 and 15 weeks of gestation, necessitating cautious imaging practices during this critical window. If the maternal thyroid gland is exposed to diagnostic radiation, it can be associated with slight decreases in birth weight. Regarding our patient, after the clinical examination, an X-ray in two incidents was performed to establish the diagnosis. For a 40-week pregnancy, X-rays could be performed, with the consent of the obstetrician, with lead-type protection, and the diagnosis of a middle third femoral fracture was established. It must be known, and the pregnant woman must be informed that there is little exposure of the fetus in limb radiographs (Table 2). Additionally, physicians should educate pregnant patients that direct in utero exposure to diagnostic X-rays has not been significantly linked to an increased risk of childhood leukemia, including lymphatic or myeloid leukemia [40]. C-arm fluoroscopy systems must maintain a minimum source-to-collimator distance of 12 inches to ensure safe operation. Additionally, minimizing the distance between the patient and the image intensifier is essential to reduce radiation exposure during the procedure. However, a minimum number of radiological investigations should be ordered. Precautions, like covering the abdomen and pelvis with a lead apron and minimal use of intraoperative C-arm radiation, should be followed [41].

Radiation exposure was minimal in this case for the patient, as the fetus was born before the orthopedic procedure took place.

Once a long bone fracture is diagnosed and the necessity for surgical intervention is established, maternal stabilization becomes the primary concern, followed by the selection of an appropriate anesthetic approach. The choice between general anesthesia or neuraxial techniques depends largely on gestational age and patient-specific factors. However, a review of existing literature indicates a lack of conclusive evidence regarding the preferred anesthetic modality for non-obstetric surgical procedures in pregnant patients. Nearly all anesthetics and analgesics used in pregnancy are category C [43]. Inhaled general anesthetic agents are not teratogenic at the levels given for surgery but have been associated with an increased risk of preterm labor in the second trimester. However, there can be an increased incidence of low and very low birth weight infants [44]. The choice of neuraxial anesthesia is based on the body mass index and the existence of less complex fractures because it has a limited action time. In our case from the anesthetic point of view, spinal anesthesia was appropriate and was performed with the tilting of the surgical table in the partial left lateral decubitus of the patient to prevent hypotension with inferior vena cava syndrome until the fetus was extracted during the C-section. It is considered to be safer in the left lateral position [45,46].

An alternative approach involves positioning a wedge under the right hip to optimize maternal circulation. It is important to recognize that spinal and epidural anesthesia can lead to maternal hypotension, which may result in placental hypoperfusion and fetal distress. To assess potential intraoperative impairments in uteroplacental blood flow and fetal oxygenation, fetal heart rate (FHR) monitoring can be utilized [47]. However, existing studies indicate that no significant changes in fetal outcomes have been definitively linked to the use of intraoperative FHR monitoring during orthopedic procedures [48].

The type of implant used for femur fractures can be either a plate or an intramedullary nail. The type of implant used affects radiation exposure. There is evidence that fractures operated on with plates compared to intramedullary nailing require decreased radiation doses [49].

The primary goal in fracture fixation should be to use the fixation technique that requires the least amount of radiation without compromising fracture care. Minimally invasive percutaneous plating techniques are commonly used. However, these difficult techniques often require high cumulative radiation exposure, which should be avoided in pregnant patients. Intramedullary nailing of comminuted long bone fractures is another example of a difficult technique. Reduction before nail insertion can be challenging, and ensuring proper alignment of the guidewire potentially increases the radiation exposure time. When the risk of radiation exposure becomes problematic, open plating techniques that involve minimal irradiation should be considered. For our patient, the orthopedist opted for closed reduction and osteosynthesis with intramedullary nail because the radiation exposure did not matter considering that the fetus had previously been evacuated by C-section.

PLO is one potential indication for a non-obstetric cause-related C-section, and a decision should be carefully taken because these women have an increased risk of spine or hip fracture. There is a lack of comprehensive data regarding the preferred mode of delivery in pregnant patients who have sustained lower limb fractures. However, in cases of pelvic fractures, the cesarean section (C-section) rate is reported to be 62%, likely due to impaired hip abduction, which may hinder vaginal delivery. Despite this trend, a case report documented a successful term vaginal delivery following surgical fixation of an acetabular femoral fracture [46]. Similarly, a retrospective case series of eight patients who delivered at late-preterm or term found that 50% underwent C-sections, though their obstetric history was not detailed [50]. Additionally, Harold JA et al. described three cases of vaginal delivery following femur fracture surgery, underscoring the importance of early postoperative physical therapy with a focus on hip abduction rehabilitation [6]. In our case, the decision for the C-section was necessary because the age of gestation was 40 weeks. The suspicion of PLO in our case was based on the combination of clinical factors, including the patient’s history, the occurrence of a low-energy fracture at term pregnancy, and the absence of other significant risk factors for pathological fractures. While we recognize that definitive confirmation would require BMD measurement, our working diagnosis was guided by previously reported cases with similar presentations.

Several experts emphasize the critical need for prompt intervention in managing fractures during the third trimester of pregnancy, as timely treatment can reduce maternal and fetal morbidity and mortality [9]. In the present case, surgical intervention was performed within the first 24 h post-injury to mitigate potential complications and optimize both maternal and fetal outcomes.

Definitive orthopedic fixation is often associated with significant postoperative and injury-related pain, necessitating the use of analgesics and muscle relaxants for patient comfort. However, pain management in pregnant patients presents unique challenges due to concerns about fetal safety. The risk of neonatal respiratory depression primarily arises when opioid analgesics are administered immediately before delivery. Recent studies indicate that epidural anesthesia provides superior pain control compared to systemic medications, suggesting that a combination of spinal and epidural anesthesia may be the preferred approach [51].

Regarding muscle relaxants, no evidence links their use to teratogenic effects, as positively charged depolarizing and non-depolarizing agents do not cross the placenta [48]. However, Hart identified cyclobenzaprine as the only muscle relaxant with established safety in pregnancy [43]. Additionally, nonsteroidal anti-inflammatory drugs (NSAIDs) are contraindicated beyond 31 weeks of gestation due to their potential to affect the ductus arteriosus, making acetaminophen the recommended alternative for mild pain management. Gestational age is a crucial determinant of medication safety, and clinicians must be aware that drug risk classifications may change throughout pregnancy, necessitating careful selection and monitoring of pharmacological interventions.

Postoperative complications can include wound hematoma, wound-edge necrosis, or surgical site infections; however, in this case, no such complications were observed. Among the most serious concerns following surgery are thromboembolic events, which require proactive prevention strategies. Pregnancy itself, along with cesarean delivery, represents a significant risk factor for venous thromboembolism (VTE) [52]. Additionally, orthopedic trauma and subsequent surgical intervention often necessitate prolonged immobilization, further elevating the risk. The incidence of VTE in patients with lower limb injuries immobilized for at least one week without thromboprophylaxis has been reported to range between 4.3% and 40% [53].

While unfractionated heparin (UFH) is traditionally used for thromboprophylaxis, it is associated with potential adverse effects, including heparin-induced thrombocytopenia (HIT), bleeding, and osteopenia. Long-term UFH therapy may lead to bone density reduction, predisposing patients to osteopenia or osteoporosis, with a reported incidence of up to 30% [54,55]. Due to these concerns, low-molecular-weight heparin (LMWH) in the form of Enoxaparin (0.4 mL, twice daily) was selected for thromboprophylaxis in this case. If delivery occurs after orthopedic surgery, LMWH administration should be discontinued 24 h prior to facilitate the safe use of epidural or spinal anesthesia and to minimize peripartum bleeding complications [56]. Following cesarean section, anticoagulation should be resumed within 12–24 h, depending on postpartum bleeding from the placental bed.

Another potential thromboprophylactic alternative is fondaparinux, a synthetic antithrombotic agent that binds selectively to antithrombin. Fondaparinux has demonstrated comparable efficacy to LMWH without the associated negative effects on bone metabolism [55]. Studies have further indicated that fondaparinux may be superior to enoxaparin, as it is associated with a significant reduction in thrombotic events and bleeding complications [57].

For high-risk cases, a more invasive strategy involves the temporary placement of inferior vena cava (IVC) filters to prevent pulmonary embolism. When positioned suprarenally, these filters can be safely removed postpartum, with no reported adverse events [58,59,60].

Bone density typically replenishes within a year postpartum, and temporary reductions in BMD during this period should not be a cause for immediate concern. In lactating women, the bone restoration process is delayed until weaning occurs, at which point bone remodeling resumes. When undergoing bone density assessments (DXA scans) within 12 months postpartum or after weaning, patients should inform their healthcare provider, as transient BMD reductions are expected. A follow-up DXA scan is generally recommended after one year to evaluate whether bone strength has returned to baseline levels [61,62]. Taking these into consideration, we recommended the patient perform DXA after surgery and again after 1 year. Studies demonstrate that bone mass density (BMD) is a better predictor of screw purchase during surgery than bone age itself [63]. Without performing DXA scanning before or after fracture fixation, the BMD could not be established [64].

## 4. Conclusions

Managing lower limb trauma in pregnant patients presents a unique clinical challenge, as treatment must prioritize the well-being of both the mother and the fetus. The complexity of orthopedic trauma during pregnancy necessitates a multidisciplinary approach, involving trauma surgeons, obstetricians, neonatologists, anesthesiologists, and radiologists, to ensure optimal outcomes. Key considerations include appropriate maternal positioning, judicious use of C-arm fluoroscopy, and strict radiation dose management to minimize fetal exposure. Diagnostic imaging for surgical planning is generally safe, provided the cumulative radiation dose remains below 5 rad.

Perioperative pain management strategies, including opioid use when necessary, can be employed with minimal risk to the fetus. Following surgical intervention, venous thromboembolism (VTE) prophylaxis with low-molecular-weight heparin (LMWH) is essential, alongside early mobilization and structured physical therapy, with a focus on hip abduction rehabilitation. Additionally, postoperative supplementation with vitamin D and calcium supports bone health and recovery.

Raising awareness among healthcare providers and patients regarding the safety and necessity of orthopedic interventions during pregnancy is crucial in reducing misconceptions and anxiety. Finally, long-term follow-up with an endocrinologist is recommended for bone mass assessment, particularly in cases where pregnancy-associated osteoporosis (PLO) is suspected. Further research is warranted to refine evidence-based guidelines for the optimal management of orthopedic trauma in pregnancy.

## Figures and Tables

**Table 1:** Recommendations for bone health.

Focus On	Recommended
Calcium	1000 mg per day from the diet. If dietary intake is low, a supplement may be required.
Vitamin D	Limited sun exposure—in summer, a few minutes per day, in winter slightly longer. Avoid UV index above 3. If vitamin D deficiency is confirmed by the doctor, a supplement may be required.
Exercise	Specific mix of weight-bearing, resistance training, and balance exercises.

**Table 2:** Radiation exposure doses for orthopedic X-rays [42].

Procedure	Fetal Exposure 1 rad = 0.01 Gy
Chest X-ray (anteroposterior/lateral)	0.02–0.07 mrad
Abdominal plain X-ray	100 mrad
Hip X-ray (single view)	200 mrad
Head or chest CT	<1 rad
Abdomen and lumbar spine CT	3.5 rad
Pelvis CT	0.25–1.5 rad
Anteroposterior pelvis	0.04 rad
Complete spine series	0.37 rad

## Data Availability

Data are available on personal request.
